# Painless Childbirth in Twilight Sleep
**Painless Childbirth in Twilight Sleep.* By Hanna Rion. London: T. Werner Laurie, Ltd. 1915. Pp. 246. Price 6s. net.


**Published:** 1915-05-08

**Authors:** 


### PAINLESS CHILDBIRTH IN TWILIGHT SLEEP.*

THE attractive and poetical title "Twilight Sleep" has recently been making its appearance in the lay Press. It is applied to a condition of diminished consciousness and sensitiveness produced in women during labour by the hypodermic injection of regulated doses of scopolamine and morphine. The name owes its origin to Gauss, of Freiburg, who, though not the first tc originate the practice, was the first to study and adopt it on the large scale. His writings on the subject attracted attention and led to some acute controversies. Not unnaturally, in view of the nature of the topic, stories appeared in various newspapers and largely took on the picturesque and sensational form which the lay reporter, knowing his public, loves to cultivate. In certain circles the thing became a fashion and a cult. Enthusiasm decorated the practice as is its wont, and anyone who hinted at difficulties and possible dangers fell under condemnation as ignorant and opposed to progress. Ladies in an interesting condition travelled to Freiburg from remote parts of the world in order that they might enjoy the advantages of painless delivery, and in various places institutions were organised to carry out the treatment as adopted in the original Frauenklinik confinement wards. If some of the incidents associated with the practice have not been altogether seemly this has been due mainly to newspaper discussion and to the overflowing gratitude of happy mothers.

Two general statements may be made in reference to the use of scopolamine and morphine for the purpose here at issue. First it is beyond question that large numbers of women have under such influences been safely delivered without suffering, or with a mere minimum of suffering, and with no bad after-effects either to themselves or their infants. Secondly, the practice is one which cannot be undertaken lightly or apart from careful study under the direction, if possible, of those experienced in the method. The question of dose is one of great delicacy, and the tests by which sufficiency or insufficiency is determined are not easy of judgment. Yet the boon, if it can be obtained, is of so welcome a nature, and the evidence that in educated hands the method is both safe and helpful is so impressive, that there seems to rest an obligation on the profession to give the matter a greater measure of attention than it appears as yet to have received. On this point it is sufficient to quote Sir Halliday Croom, the well-known Professor of Midwifery in the University of Edinburgh, who writes so recently as February last, "Ever since its [scopolamin-morphin] first introduction I have used it regularly in every private case under my care, rendering the whole process a dream, and without the slightest bad effects either to the mother or the child. Sometimes the baby was a little drowsy, but was easily roused." This considered judgment must be treated with respect, and it has received support from other British writers on the subject. Here at least are authorities quite beyond challenge.

On a different plane, but by no means to be disregarded, is a recently published book on the subject, and addressed quite frankly to the "great mother-public." The writer, Mrs. Rion, has accepted her self-imposed mission in a very serious and earnest spirit, and though she sometimes ruffles our professional susceptibilities, and perhaps other delicacies also, we must not fail to pay a tribute to her industry and enthusiasm. Her ambition is to inform and to stimulate the members of her sex so that these may bring pressure to bear on the doctors. Medical discussions and medical journals are all very well in their way, but the crying need is to reach the mothers themselves. Even the communication of the blessing "by mouth propaganda" is at best a slow process, and in view of all these hindrances there has been no other method than the writing of a book intended to spread the good news. With the suggestion that the choice of an anæsthetic must rest with the doctor Mrs. Rion finds it difficult to be patient; this professional conservatism must be overcome by an informed public opinion which may be counted on to quicken the natural sluggishness of the medical mind. Whether this is quite the best method of proceeding we will not for the moment stop to inquire, but we certainly doubt whether Mrs. Rion's plan of campaign is likely to be highly successful. In her extensive study of the question she has entered into all the discussions, and she gives full translations of the opponents' papers and speeches as well as of those on her own side. It is true that she devotes herself to the task of showing how wrong either in head or in technique the opponents are, but we fancy that an expectant mother reading her book would long before she reached the last page have a bewildered sense that really the proceeding proposed was not altogether free from risk. Nor do we think that a photograph of a Scottish maternity hospital or the announcement that one of the successful ladies was a "most splendid creature of the Wagnerian type" is likely to have a reassuring effect. It may be quite true that every woman physician whom Mrs. Rion has met is "altogether adorable," and that Mrs. Stammnitz "after a forty-eight hours' birth" wrote a poem of four pages as a proof that her mental faculties were not dulled by the drug, but these touching incidents are likely to be neutralised in the mind of anxious women by a reference to the possibility of atonic hæmorrhages, to kolpoporexis, and to oligopnœic, apnœic and asphyxtic infants! In short, so far as the public is concerned, Sir Halliday Croom's letter is likely to be worth more than all Mrs. Rion's arguments and statistics and translations. We cannot suggest she should get rid of all these, for that would be unkind. But she certainly would be wise to get rid of the covering letter which the publisher encloses with her book.

---

* *Painless Childbirth in Twilight Sleep.* By Hanna Rion. London: T. Werner Laurie, Ltd. 1915. Pp. 246. Price 6s. net.

